# Genome-wide identification and characterization of tissue specific long non-coding RNAs and circular RNAs in common carp (*Cyprinus carpio* L.)

**DOI:** 10.3389/fgene.2023.1239434

**Published:** 2023-11-28

**Authors:** Jutan Das, Baibhav Kumar, Bibek Saha, Sarika Jaiswal, Mir Asif Iquebal, U. B. Angadi, Dinesh Kumar

**Affiliations:** ^1^ Division of Agricultural Bioinformatics, ICAR-Indian Agricultural Statistics Research Institute, New Delhi, India; ^2^ Graduate School, ICAR-Indian Agricultural Research Institute, New Delhi, India

**Keywords:** *Cyprinus carpio*, circRNAs, circRNA-miRNA-mRNA interaction, GO, lncRNAs, lncRNA-miRNA-mRNA interaction, KEGG, RNA-seq

## Abstract

*Cyprinus carpio* is regarded as a substitute vertebrate fish model for zebrafish. A varied category of non-coding RNAs is comprised of long non-coding RNAs (lncRNAs) and circular RNAs (circRNAs). These ncRNAs were once considered non-functional “junk DNA” but research now shows they play important roles in gene expression regulation, chromatin modification, and epigenetic regulation. The systemic tissue-specific research of the lncRNAs and circRNAs of *C. carpio* is yet unexplored. A total of 468 raw RNA-Seq dataset across 28 distinct tissues from different varieties of common carp retrieved from public domain were pre-processing, mapped and assembled for lncRNA identification/ classification using various bioinformatics tools. A total of 33,990 lncRNAs were identified along with revelation of 9 miRNAs having 19 unique lncRNAs acting as their precursors. Additionally, 2,837 miRNAs were found to target 4,782 distinct lncRNAs in the lncRNA-miRNA-mRNA interaction network analysis, which resulted in the involvement of 3,718 mRNAs in common carp. A total of 22,854 circRNAs were identified tissue-wise across all the 28 tissues. Moreover, the examination of the circRNA-miRNA-mRNA interaction network revealed that 15,731 circRNAs were targeted by 5,906 distinct miRNAs, which in turn targeted 4,524 mRNAs in common carp. Significant signaling pathways like necroptosis, NOD-like receptor signaling pathway, hypertrophic cardiomyopathy, small cell lung cancer, MAPK signaling pathway, etc. were identified using Gene Ontology and Kyoto Encyclopedia of Genes and Genomes. The web resource of common carp ncRNAs, named *CCncRNAdb* and available at http://backlin.cabgrid.res.in/ccncrnadb/ gives a comprehensive information about common carp lncRNAs, circRNAs, and ceRNAs interactions, which can aid in investigating their functional roles for its management.

## 1 Introduction

The deluge of next-generation sequencing data due to advanced high-throughput technology has facilitated the genome-wide identification of RNAs in various species, leading to the discovery of multiple non-coding RNA (ncRNAs) genes (Rinn and Chang, 2012; Kung et al., 2013). The ncRNAs are heterogeneous sets of RNA molecules that do not undergo protein translation (Alexander et al., 2010). Long non-coding RNAs (lncRNAs) are a class of ncRNA molecules with lengths >200 nucleotides (nts) that lack an identifiable open reading frame (ORF) and a conserved codon suggests that there is no possibility for protein-coding (Novikova et al., 2013). Initially lncRNAs were believed to be an insignificant by-product, produced during gene transcription by RNA polymerase II, with no biological purpose other than generating “noise genes”. However, a study has revealed their involvement in regulating mammalian X chromosome inactivation, leading to further exploration of non-coding RNAs (Brown et al., 1992; Lee et al., 1993).

Global gene expression data from various mammalian species, reveals that <2% of the genome comprises protein-coding sequences, while the remaining is transcribed into non-coding RNAs (Clark et al., 2011; Djebali et al., 2012; Berthelot et al., 2014). In recent years, there has been a surge in research interest in lncRNAs due to their potential involvement in regulating various biological processes through transcriptional or post-transcriptional regulatory mechanisms (Batista and Chang, 2013). LncRNA has categorized into three main types, namely, intergenic, intronic, and exonic. Intergenic lncRNAs originate from the intergenic regions, while intronic lncRNAs are solely derived from introns, and exonic lncRNAs are derived from exons of protein-coding genes (Mercer et al., 2009). The identification and annotation of lncRNA sequences are challenging due to their lower expression levels and less conserved nature, setting them apart from small non-coding RNAs and posing computational difficulties (Derrien et al., 2012). LncRNAs are recognized as significant gene regulators owing to their roles as decoys, scaffolds, or guides. By blocking regulatory proteins’ access to DNA, these lncRNAs influence the transcription of protein-coding genes (Kino et al., 2010; Hung et al., 2011). In addition to extensive studies conducted in humans, other mammals, and plants, various lncRNAs have also been identified in fish species including zebrafish (Pauli et al., 2012; Chen et al., 2018), coho salmon (Leiva et al., 2020), rainbow trout (Al-Tobasei et al., 2016), large yellow croaker (Jiang et al., 2016; Zhang et al., 2020), tilapia (Li et al., 2018), common carp (Song et al., 2019; Wang et al., 2021; Hu et al., 2023), koi carp (Luo et al., 2019; Yang et al., 2022), black carp (Zhang et al., 2022), bighead carp (Fu et al., 2019), grass carp (Gan et al., 2020), and amur carp (Zhao et al., 2021).

Besides the linear lncRNAs, a significant group of non-linear ncRNAs known as circRNAs (circular RNA) has arisen. CircRNAs are variants of transcripts that arise from unconventional splicing, whereby the RNA is circularized through the formation of covalent bonds between the 5′ donor end and 3′ acceptor junctions through back splicing (Salzman et al., 2012). In the 1970s, plant viroid and hepatitis delta virus were the first to be recognized as containing circRNA (Sanger et al., 1976; Kos et al., 1986). CircRNAs can control the expression of their linear counterparts by limiting the pre-mRNA available for traditional splicing, operating at a functional level (Salzman et al., 2012). The circRNA-related research has been carried out in zebrafish (Shen et al., 2017; Sharma et al., 2019; Ranjan et al., 2021), rainbow trout (Wu et al., 2022), tilapia (Fan et al., 2019), Japanese flounder (Ning and Sun, 2021b), golden pompano (Sun et al., 2023), and large yellow croaker (Xu et al., 2017).

As per SDG14, conservation and the sustainable use of ocean-based resources should be prioritized. SDG14 aims to conserve and sustainably use the oceans, seas and marine resources for sustainable development (https://sdgs.un.org/goals/goal14). The issue of overfishing can be controlled by improving productivity. In such approach, role of lncRNA-miRNA-mRNA axis has immense impact on aquaculture productivity, fish health, and quality (Zhou et al., 2023). Besides this, lncRNA has key role in immunity of fish which is directly linked with productivity (Haridevamuthu et al., 2023). Aquaculture is currently considered essential in ensuring food security and maintaining economic stability, and it is the most rapidly expanding farmed food industry on a global scale due to the depletion of natural fish populations (Jennings et al., 2016). Common carp (*Cyprinus carpio*) is a highly significant edible fish species that exist in over a hundred strains and forms worldwide (Balon, 1995; Teletchea, 2015). The freshwater portions of rivers in northern India, Bangladesh, Pakistan, and Burma are the natural habitat of Indian major carp, which are predominantly raised in those countries (Jhingran and Pullin, 1985). Even though a research provides information on the genome and genetic variety of *C. carpio* (Xu et al., 2014), but genome-wide or tissue-specific ncRNAs and interaction studies with miRNAs and mRNAs are still warranted. Here, tissue-specific means separate analysis were done for each of the 28 tissues to identify the lncRNAs and circRNAs. In this study, we aim at identification and characterization of lncRNAs and circRNAs in the common carp genome, collected from 28 tissues, establishing ceRNAs network involving lncRNA/circRNA-miRNA-mRNA, functional roles of genes and development of the first web-based database of common carp non-coding RNA database, *CCncRNAdb*.

## 2 Materials and methods

### 2.1 Data collection

For the study, a total of 468 raw RNA-Seq datasets were obtained from the National Center for Biotechnology Information (NCBI) database (https://www.ncbi.nlm.nih.gov/) for common carp, covering >9.7 billion transcript reads. The dataset encompassed 42 bioprojects from 23 institutions across 7 countries. These included data across 28 distinct tissues from different varieties of common carp (viz. *koi*, *haematopterus*, *specularis*, *color*, *huanghe*, *wuyuanesis*, *singuonensis*, *jian*), the details of which are provided in Supplementary Table S1. All the bioinformatics analysis were tissue-specific, i.e., performed separately for each of the 28 tissues.

### 2.2 Data quality analysis, mapping, and transcriptome assembly

The raw reads obtained from the NCBI were first visually assessed for the quality using *FastQC* tool ver. 0.11.8 (Schmieder and Edwards, 2011) followed by elimination of the adaptor sequences and low-quality reads using *Trimmomatic* software ver. 0.39 (Bolger et al., 2014). To ensure non-interference of polyA tail, reads were trimmed off with primer, followed by automatic transcriptome assembly. Moreover, lncRNAs are identified based on alignment with reference sequence which further ensures exclusion of such tails as they are not genetically coded. They are added post-transcriptionally for protection/shelf life regulating translational efficiency. Common carp reference genome and annotation files were downloaded from NCBI (https://www.ncbi.nlm.nih.gov/assembly/GCF_000951615.1/) (Xu et al., 2014). Index files of the reference genome were generated using the HISAT2-build function of HISAT2 version 2.2.0 (Kim et al., 2015; Pertea et al., 2016). Sam files were aligned and converted to binary bam files using Samtools software version 1.9 (Li et al., 2009). StringTie software version 2.1.4 was used for transcriptome assembly of the individual bam files and generate Gene Transfer Format (gtf) files for each transcriptome reads (Pertea et al., 2015; Pertea et al., 2016). Finally, the StringTie-merge feature was used to combine tissue-specific files and generate a single gtf file per tissue (Banerjee et al., 2021).

### 2.3 Identification of lncRNAs in common carp

**FIGURE 1:**
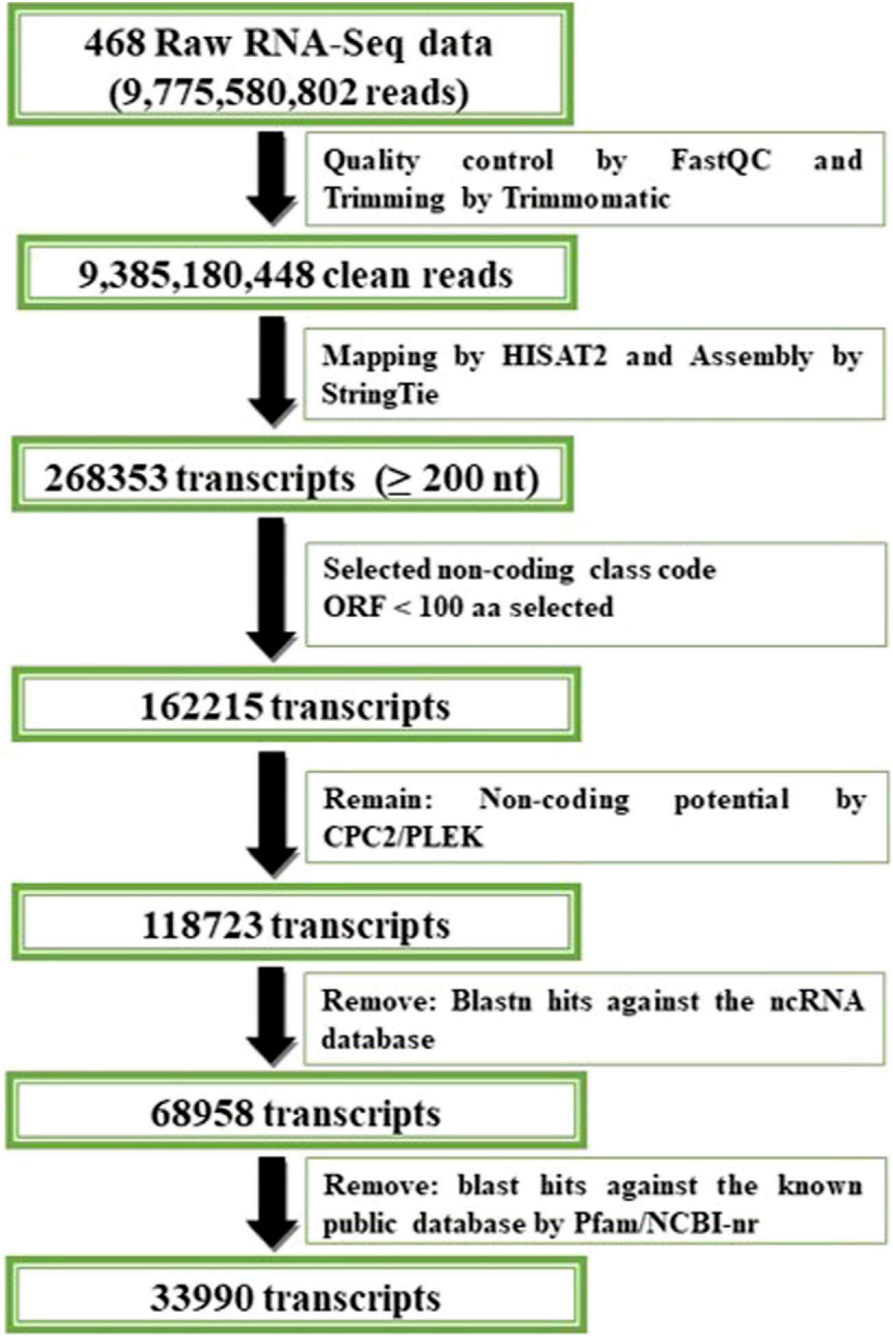
Pipeline for identification of lncRNA in *C. carpio*.

To identify potential lncRNA transcripts, the FASTA sequences that corresponded to every transcript within the combined assembly file were obtained by the *gffread* program version 0.12.3 using the respective reference genome (Pertea and Pertea, 2020). Owing to the longer size of lncRNAs, transcripts <200 base pairs were removed using perl scripts. ORFs were predicted using ORFPredictor, and those exceeding 300 nucleotides were eliminated (Min et al., 2005; Wang et al., 2021). The coding potential of the transcripts were assessed using CPC2 ver 1.0.1 (Kang et al., 2017), and PLEK ver 1.2 (Li et al., 2014), eliminating the coding RNAs. Non-coding RNAs were discovered through a BlastN search against RNACentral (https://rnacentral.org/) and transcripts with at least 95% identity were excluded (Shumayla et al., 2017). The remaining transcripts might contain small classes of non-coding RNAs, such as mRNA, tRNA, rRNA, miRNA, and snRNA. These underwent Blastp search (Mount, 2007) against the Pfam (http://pfam.xfam.org/) database (Altschul et al., 1990) and non-redundant database (https://www.ncbi.nlm.nih.gov/protein/) to eliminate recognized protein-coding RNAs according to the *C. carpio* annotation. The remaining transcripts were considered to be the potential lncRNAs for further analysis. The FPKM values for these transcripts were also calculated. The pipeline for identification of lncRNA in *C. carpio* is delineated in Figure 1.

### 2.4 Classification of lncRNAs

The *gffcompare* software was used to categorize lncRNAs into different groups based on their position relative to protein-coding genes, *namely*, lincRNA (intergenic lncRNAs), labeled as “u”; exonic lncRNAs (intersecting with protein-coding exons), labeled as “x”; and intronic lncRNAs (existing in introns without sharing sequences with exons), labeled as “i” (Leiva et al., 2020). Further, the protein-coding genes were eliminated through an evaluation and elimination process.

### 2.5 Conservation analysis of lncRNAs

Compared to protein-coding mRNAs in different species, lncRNAs have lower conservation (Derrien et al., 2012). The conservation of lncRNAs in *C. carpio* with zebrafish, rainbow trout, and large yellow croaker was assessed using BLAST, with a cutoff E-value < 1e^−6^ (Zhang et al., 2022). Zebrafish data were obtained from the ZFLNC database (https://www.biochen.org/zflnc/) (Hu et al., 2018), while information on rainbow trout and large yellow croaker came from previous lncRNA profiling studies (Al-Tobasei et al., 2016; Zhang et al., 2020).

### 2.6 Characterization and functional annotation of lncRNAs and circRNAs

#### 2.6.1 Interaction between lncRNAs and miRNAs

The relationship between lncRNAs and miRNAs is complex and intricate. Previous studies have suggested that lncRNAs might enhance pri-miRNA processing or serve as precursors for miRNAs (Augoff et al., 2012; Jiang et al., 2017). Through endonuclease activity, pre-miRNAs are cleaved to produce mature miRNAs, typically 18–24 nucleotides long. To determine if *C. carpio* lncRNAs act as miRNA precursors, the identified lncRNA sequences were aligned with miRNAs from the miRBase database using Blastn, aiming to identify any known miRNA precursors. The Vienna RNA package within the RNAfold program was utilized to predict the secondary structures of lncRNA transcripts (Leiva et al., 2020).

#### 2.6.2 Construction of lncRNA-miRNA-mRNA interaction network

The microRNA target prediction tool psRNAtarget (V2, 2017 release) (http://plantgrn.noble.org/psRNATarget/) (Dai and Zhao, 2011) was used to carry out an analysis of the interactions between lncRNA-miRNA and miRNA-mRNA (Zhou et al., 2021; Khan et al., 2022). Identified common carp lncRNAs corresponding miRNA were taken from previous studies (Wang et al., 2017a) while the mRNAs data were retrieved from NCBI database. The parameters considered were the expectation ≤2, disallowance of bulges/gaps, and a maximum unpairing energy (Max UPE) up to 25. The ceRNAs (lncRNA-miRNA-mRNA) interaction network was then constructed and visualized by combining the lncRNA-miRNA network and the miRNA-mRNA network using Cytoscape (Shannon et al., 2003).

#### 2.6.3 Identification of circular RNAs (circRNAs) in common carp genome

**FIGURE 2:**
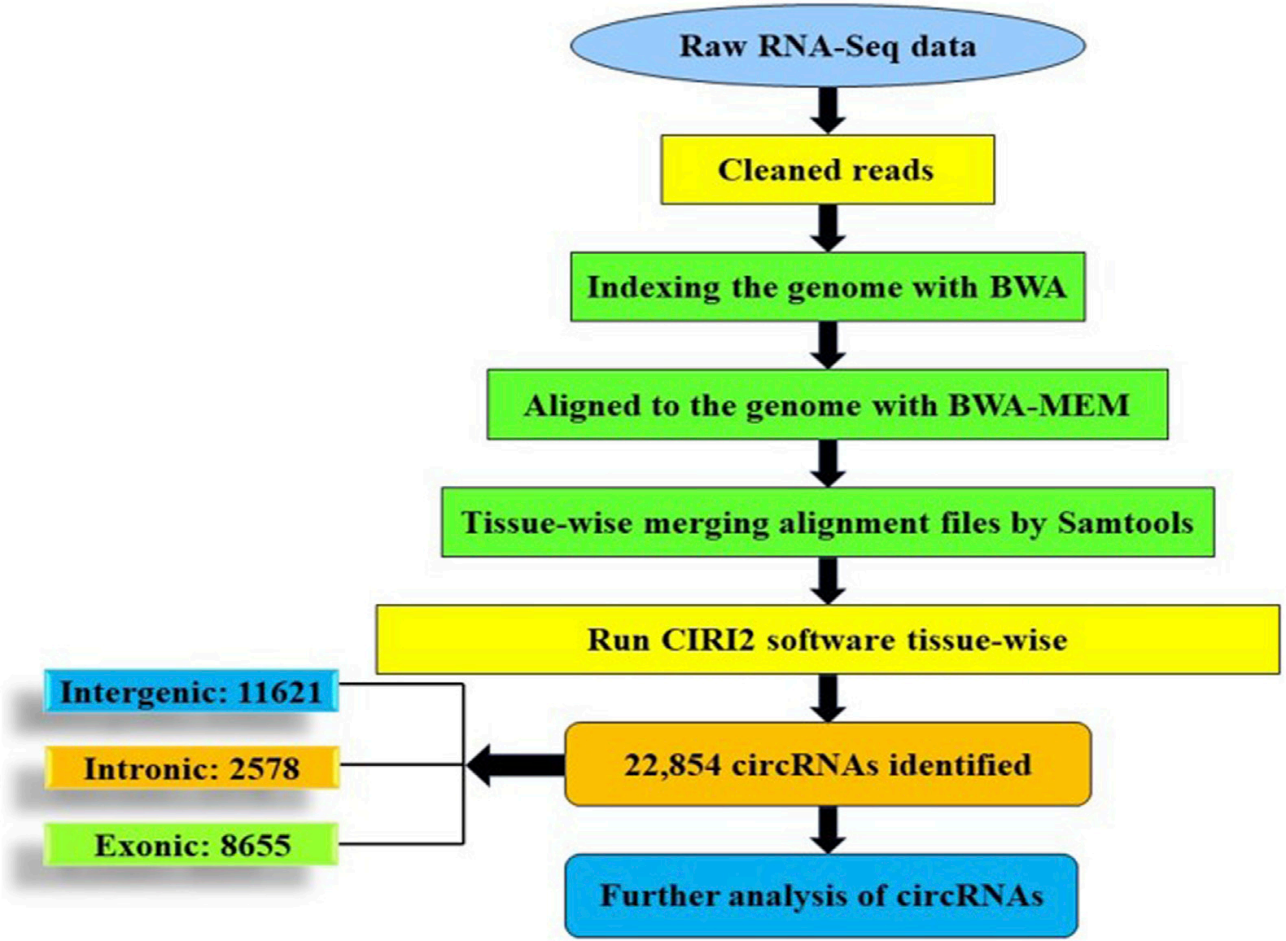
Schematic representation of circRNA identification.

For identification of circular RNAs, CIRI2 circRNA identification pipeline was used. Raw reads from the NCBI database were evaluated using FastQC, followed by Trimmomatic to remove contaminants and low-quality reads. BWA software version 0.7.17 was used to align clean reads to the common carp reference genome. An index was created using the BWA -index module before alignment, which was done using BWA mem -T 19 (Li, 2013). SAM files of each tissue were merged using Samtools version 1.14 (Li et al., 2009). The merged SAM files were used for circRNA identification in common carp using CIRI2 tool (version 2.0) with default parameters (Gao et al., 2018) (Figure 2).

#### 2.6.4 Construction of ceRNAs interaction (circRNA-miRNA-mRNA) network

A ceRNAs network was constructed based on the ceRNAs theory, specifically the circRNA-miRNA-mRNA network, in order to gain insight into the interactions among circRNAs, miRNAs, and mRNAs. The miRNA dataset for common carp was obtained from literature (Wang et al., 2017b), while the mRNA data was acquired from the NCBI database. TargetFinder was used to identify circRNA targets of miRNAs (Fahlgren and Carrington, 2010). MRNA targets of identified miRNAs were determined by submitting miRNA sequences and common carp mRNA sequences to the psRNAtarget webserver.

#### 2.6.5 GO and KEGG analysis

GO and KEGG analyses were performed using annotation results for common carp. However, directly usable GO and KEGG data for carp is currently unavailable (Wang et al., 2021). To understand the roles of host genes for identified lncRNAs and circRNAs, parent genes underwent GO and KEGG enrichment analyses. Gene IDs and total gene count were extracted and functionally annotated using Blast2GO (Conesa et al., 2005), which served as the background for GO and KEGG enrichment analyses using http://geneontology.org/ and http://www.genome.jp/kegg/, respectively (Xu et al., 2017).

#### 2.6.6 Web resource for ncRNAs of *C. carpio*

A web resource in the form of common carp non-coding RNA database (*CCncRNAdb*) was developed for significant impacts of research in this field by providing a valuable resource, promoting collaboration, and enhancing data accessibility. This is based on three-tier architecture viz., client, middle, and database tiers. The database tier utilizes MySQL for storage information related to tissue-specific lncRNAs, circRNAs, their interactions with miRNAs and mRNAs, and miRNA-mRNA interactions for both lncRNAs and circRNAs. The web interface was designed using PHP and HTML, and enhanced with CSS and JavaScript to make it dynamic. The database was hosted on an Apache server and XAMPP was used for webpage design and deployment. Data retrieval involves user requests, MySQL queries, database responses, and server-user communication.

## 3 Results

### 3.1 Overview of RNA-seq data, reads mapping and transcriptome assembly results

A total of 9,775,580,802 raw reads generated by Illumina HiSeq platform from 28 different tissues was collected from 468 RNA-seq datasets from the NCBI SRA database. After discarding adaptor sequences and low-quality reads, 9,385,180,448 clean reads (94.88%) were obtained. Approximately 80% of the clean reads from the 468 samples were aligned to the reference genome of the common carp using HISAT2 (Supplementary Table S2). A total of 268,353 transcripts for tissue-specific lncRNAs were generated using StringTie-merge module.

### 3.2 Genome-wide identification of lncRNAs of *C. carpio*

**FIGURE 3:**
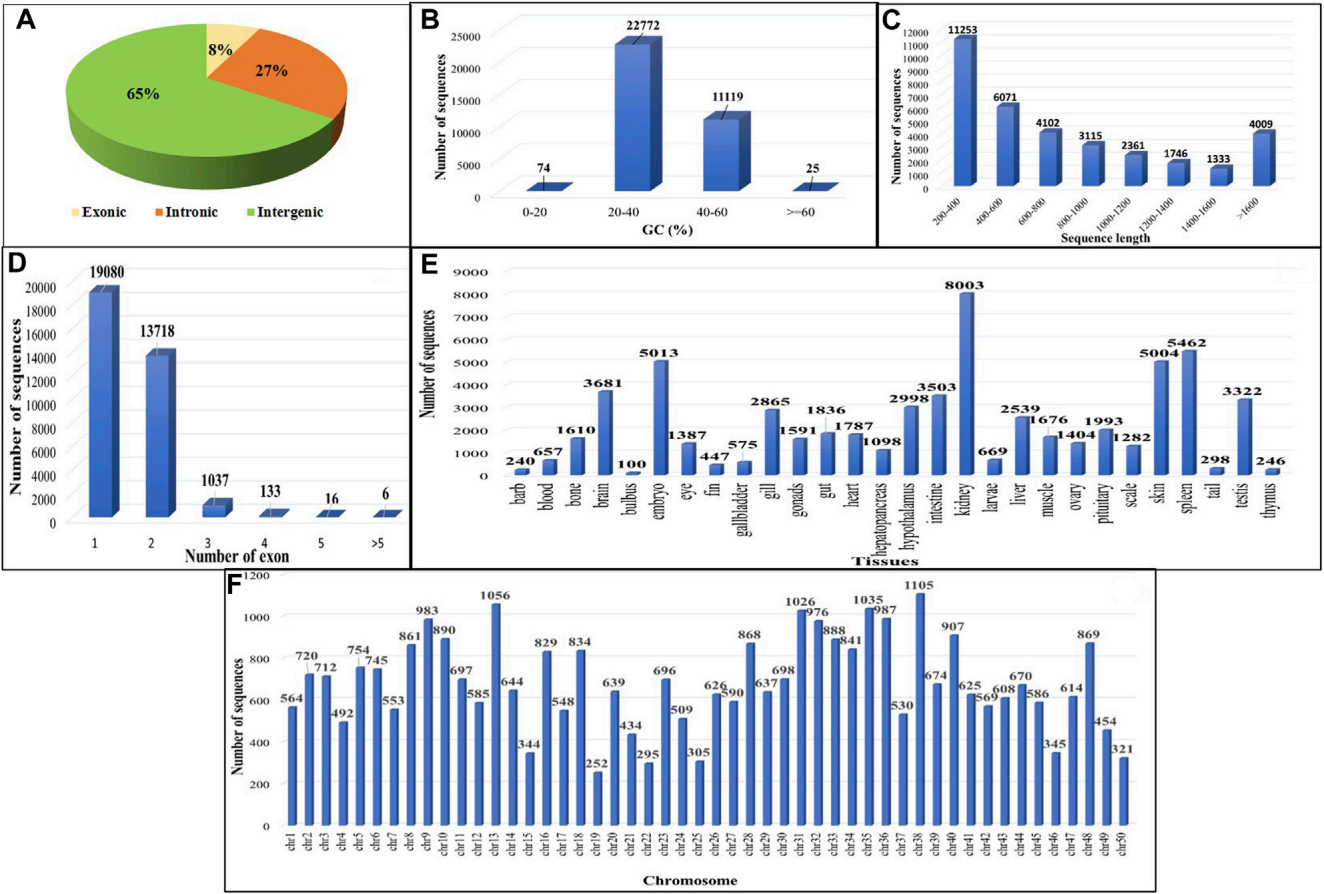
Features of common carp lncRNA **(A)** Distribution of lncRNA subtypes **(B)** Common carp lncRNAs GC content distribution **(C)** Distribution of common carp lncRNA length **(D)** Distribution of common carp lncRNA exon number **(E)** Tissue wise distribution of common carp lncRNAs **(F)** Common carp lncRNA distribution in chromosomes.

On comparing the four GTF files to the existing *C. carpio* annotation file using GffCompare, allowing transcript annotation based on genomic location relative to known genes, 300,218 transcripts were found to lie in the u (unknown intergenic), x (genic antisense) and i (intronic) classes (Figure 3A). A total of 33,990 putative lncRNAs were discovered in *C. carpio* after applying filtering criteria, namely, eliminating nucleotide sequences <200 nucleotides, ORF length >300 nucleotides, CPC2 score >0.5, housekeeping RNAs with >95% identity and transcripts similar to protein families or genes. Majority of the sequences showed GC% between 20 and 40 (Figure 3B), while the most abundant sequence length was 200–400 bp, followed by 400–600 bp (Figure 3C). Almost 56.13% of sequences had one exon, followed by 40.36% having two exons (Figure 3D). The distribution of these lncRNAs across different tissues shows kidney tissues to exhibit the highest number of identified lncRNAs, i.e., 8,003 (23.54%) (Figure 3E). We found three lncRNAs, TCONS_00121934, TCONS_00177318, and TCONS_00328247, abundantly expressed in all 28 tissue types we studied. Figure 3E shows the chromosome-wise distribution of lncRNAs in common carp. The calculate FPKM value across all 28 tissues and distinct levels of expression were discerned, as revealed by the comprehensive analysis of the bioprojects. The study unveiled a diverse range of average expression values for lncRNAs across multiple tissues as provided in Supplementary Table S3.

### 3.3 Classification and conservation of identified lncRNAs in *C. carpio*

Out of the total 33,990 lncRNAs, the majority (65.30%) belonged to intergenic class of lncRNAs (u), followed by intronic lncRNAs (i) and exonic lncRNAs (x) which were 27.28% and 7.39%, respectively (Figure 3A). These findings suggest that the majority of lncRNAs (i.e., intergenic or lincRNAs), do not overlap with protein-coding genes. To investigate the conservation of lncRNAs among various species, these putative lncRNAs discovered in *C. carpio* were compared to those of zebrafish, rainbow trout, and large yellow croaker through a blast analysis. The results showed that only a small number of lncRNAs, i.e., 3,484 (10.25%) in zebrafish (cyprinid), 138 (0.4%) in rainbow trout (salmonid), and 43 (0.12%) in large yellow croaker (sciaenidae) were conserved (Supplementary Table S4). This low level of similarity may be due to poor conservation of lncRNAs across species and tissues.

### 3.4 Characterization of common carp lncRNAs

**FIGURE 4:**
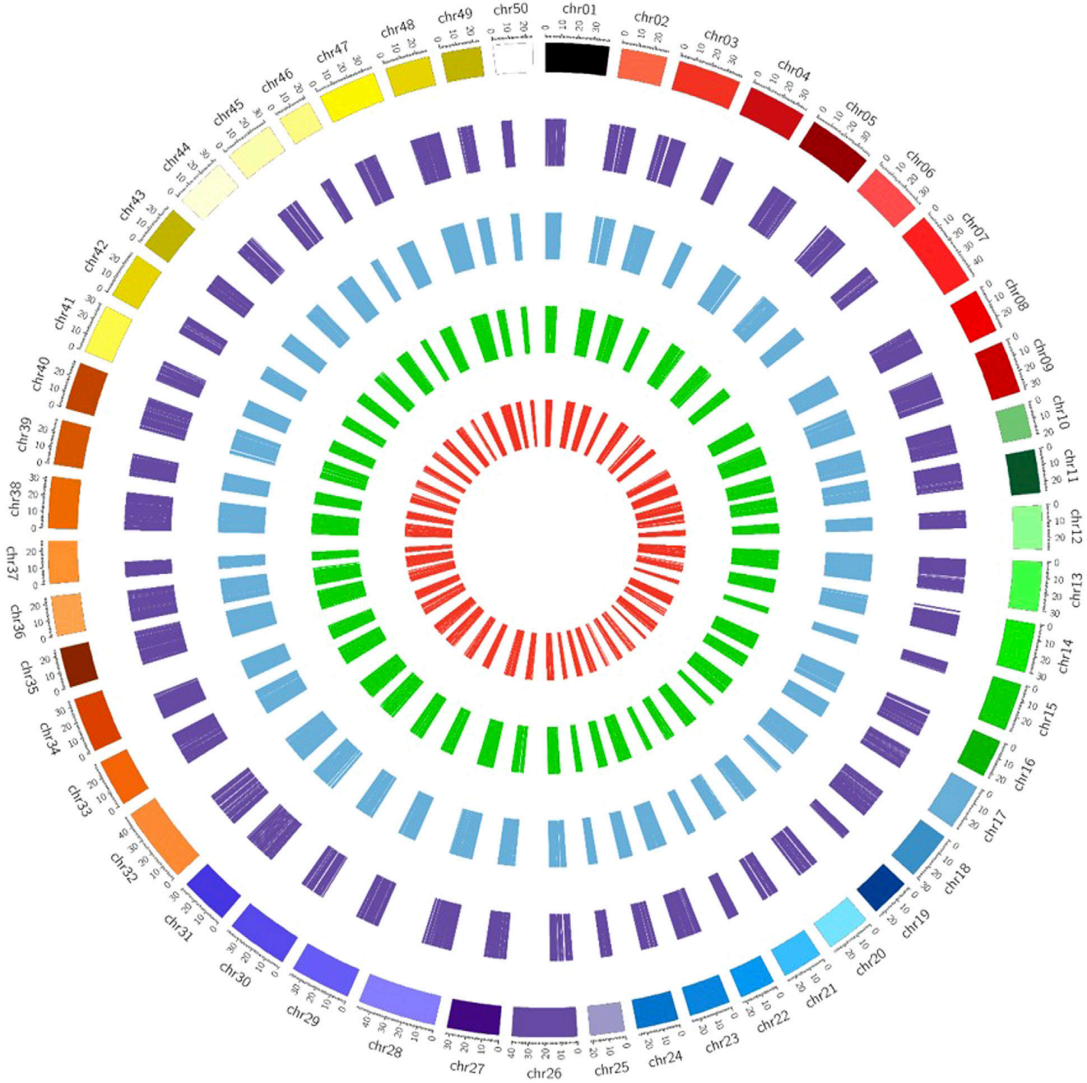
Chromosome-wise distribution of lncRNAs and circRNAs of common carp. The visual representation consists of different circles, with the outermost circle representing circRNAs (dark purple color), intergenic lncRNAs in blue color, intronic lncRNAs in dark green color, and exonic lncRNAs in the inner circle (red color).

It was observed from the analysis that the most common carp lncRNAs had a GC content ranging from 0% to 66.4%, but the majority (99.70%) were within 20%–60%, with an average GC content of 37.88% (Figure 3B). The length of lncRNAs in common carp varied from 200 to 15,799 nucleotides (Figure 3C), with 72.17% being between 0 and 999 nucleotides and 27.82% between 1,000 and 1,999 nucleotides. The average length of the lncRNAs was found to be 837 nucleotides, which is shorter than the length of protein-coding genes (2.914 kb). Common carp lncRNAs had 1–7 exons on average (1.48 exons). The majority (96.49%) were single or double exon types, with an average length of 849.82 nucleotides (Figure 3D). The chromosomal distribution of the identified lncRNA in common carp is shown in (Figure 3F), with chromosome 38 having the highest count of 1,105 lncRNAs and chromosome 19 having the lowest count of 252. This distribution was visualized using Circos software (Figure 4).

### 3.5 lncRNAs mediating miRNA function

The identified lncRNAs were analyzed for their potential target genes based on their proximity to protein-coding genes. Additionally, the sequences of the lncRNAs were compared to miRNAs from miRBase using the BlastN program to determine if they function as miRNA precursors. Results showed that 9 pre-miRNAs matched with 19 distinct lncRNAs in common carp, indicating the potential for these lncRNAs to produce mature miRNAs at matching accuracy cut-off of ≥90% (Supplementary Table S5). Few lncRNAs were discovered to have a stable hairpin structure, indicating the presence of a miRNA precursor. In our study, TCONS_00417363 lncRNA (dark green), which contained the miRNA precursor ami-mir-133a-2 (red) was identified by Vienna RNA package and RNAfold program (Supplementary Figure S1A).

### 3.6 Analysis of lncRNA-miRNA-mRNA interaction network

LncRNAs play a crucial role in regulating gene expression by acting as competitive endogenous RNAs (ceRNAs), which capture miRNAs and prevent them from binding to their target mRNAs. This interaction between lncRNAs and miRNAs significantly affects gene expression and various biological processes. The miRNAs also regulate gene expression by binding to the 3′ untranslated region (UTR) of target mRNAs, leading to mRNA degradation or translational repression. These miRNA-mediated gene expression regulations are essential for the normal development and function of fish organs and tissues. Using the psRNAtarget server, 18,977 interactions (lncRNA-miRNA) involving 2,837 distinct miRNAs and 4,782 distinct lncRNAs in common carp were identified. Using the RNAfold program with the Vienna RNA package, a visualization of the secondary structure of lncRNA TCONS_00239471 (displayed in dark green), along with the locations of its binding sites for miRNAs ccr-miR-338 (depicted in red) and mir-338-y (depicted in blue) were generated (Supplementary Figure S1B). In the case of miRNA-mRNA, a total of 8,079 interactions, which involved 1,900 unique miRNAs and 3,718 unique mRNAs were revealed. The Cytoscape software was utilized to construct individual lncRNA-miRNA and miRNA-mRNA interaction networks after that merged both the network in a single network and visualizing the whole lncRNA-miRNA-mRNA interaction network. From this interaction analysis, we observe that mir-8499-y target 2 lncRNAs of TCONS_00413548, TCONS_00007855 and six mRNAs these are lcl|LHQP01046800.1_mrna_46126, lcl|LHQP01016561.1_mrna_24063, lcl|LHQP01011828.1_mrna_17740, lcl|LHQP01021498.1_mrna_29844, lcl|LHQP01064132.1_mrna_49370 and lcl|LHQP01015883.1_mrna_23319 (Supplementary Figure S2).

### 3.7 Identification of circular RNAs in common carp

**FIGURE 5:**
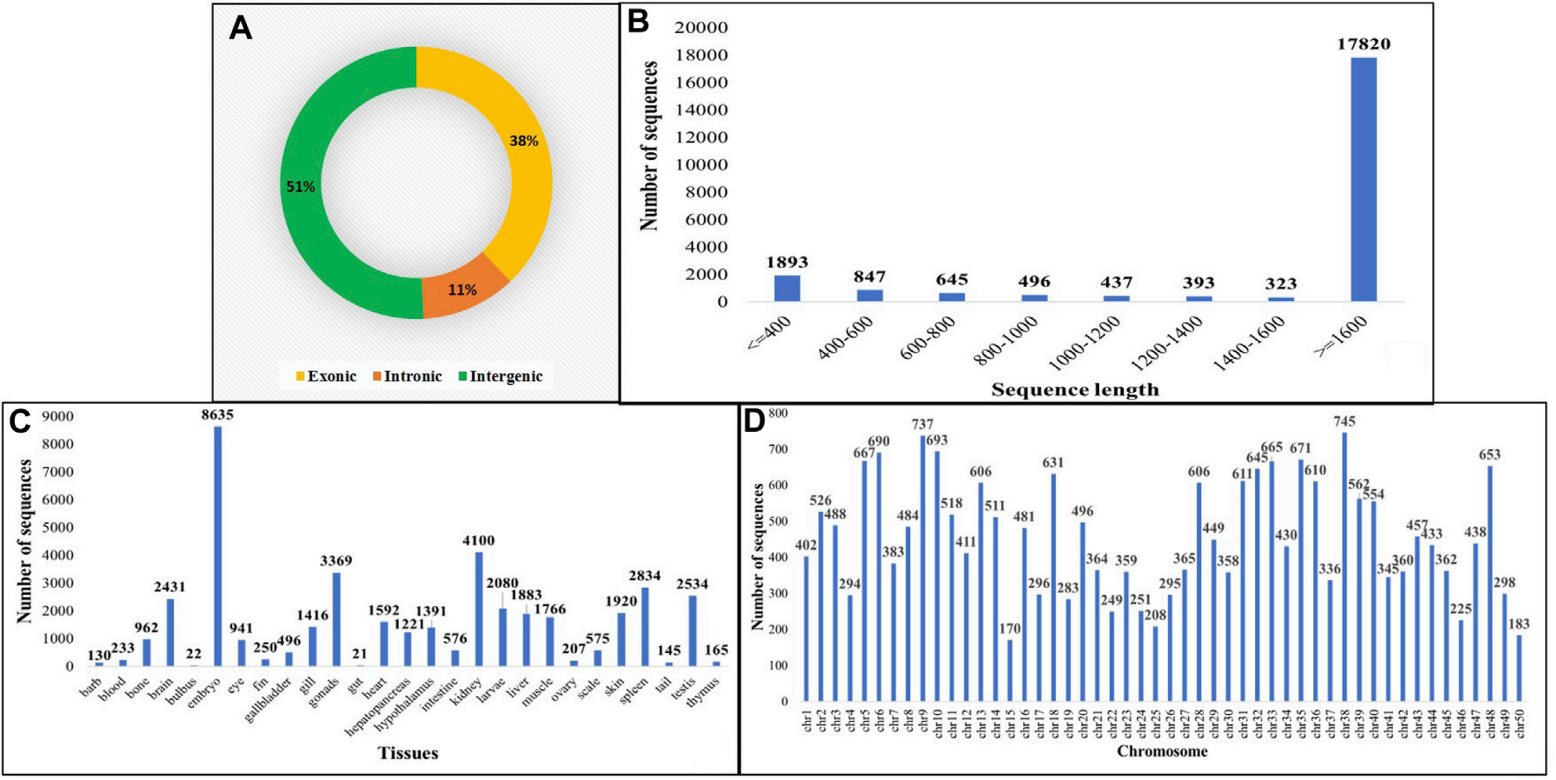
Features of common carp circRNA **(A)** circRNA subtypes distribution **(B)** Length-wise distribution of common carp circRNA **(C)** Tissue-wise distribution of common carp circRNAs **(D)** Chromosome-wise distribution of circRNA.

The identification of circRNAs in common carp involved four main step: i) quality check of 9,775,580,802 raw RNA-seq reads from NCBI ii) final clean reads aligned to the common carp reference genome using BWA-mem -T 19 ii) generation of 468 SAM files for each sample, followed by merging for each of the 28 tissues. iv) Submission of these merged files to the widely used command-line tool, CIRI2. This resulted into 22,854 distinct potential circRNAs (Supplementary Table S6). In this study, we have not only identified tissue-specific common carp circRNAs but have also uncovered striking differences in their abundance across tissues. A total of 11,621 circRNAs (50.84%) were derived from intergenic regions, while only 2,575 (11.26%) were generated by introns (Figure 5A). Out of the identified circRNAs, 8,655 (37.87%) originated from exons of protein-coding genes, indicating they were exonic circRNAs. These exonic circRNAs had both back-splice sites aligned with known exonic boundaries. A visual representation of the length distribution of circRNAs reflected that majority of circRNAs were over 1,600 nucleotides long (Figure 5B). Specifically, embryonic tissue showed the abundance of circRNAs (8635) (Figure 5C). Chromosome 38 was seen to have highest number of circRNA (745) (Figure 5D). The chromosome-wise circos-map of circular RNAs in common carp is summarized in Figure 4 (outermost dark purple circle).

### 3.8 Analysis of circRNA-miRNA-mRNA interaction network

CircRNA-miRNA-mRNA interactions have been identified as an important regulatory mechanism in gene expression which is also called the ceRNAs network, where circRNAs act as a sponge for miRNAs, thereby inhibiting the degradation of target mRNAs by miRNAs. By using TargetFinder software we get a total of 970,159 interactions (circRNA-miRNA) where 5,906 unique miRNAs were found targeting 15,731 circRNAs in the tissues under study, except the pituitary tissue. As a result, 10,484 interaction (miRNA-mRNA) was found from the psRNATarget server analysis where 4,524 distinct mRNAs were targeted by 2,871 unique miRNAs of common carp. An entire circRNA–miRNA-mRNA interaction network was delineated by Cytoscape (Supplementary Figure S3). The figures show that miRNA, mir-6627-y targeted a total of seven mRNAs, miRNA mir-6651-x targeted nine circRNAs and two mRNAs, while another miRNA, mir-7371-x targeted a total of 13 circRNAs.

### 3.9 Functional annotation of common carp lncRNAs and circRNAs

In this study, we utilized GO and KEGG annotations to gain understanding of the functions of lncRNAs/circRNAs, based on the hypothesis that their functions may be linked to those of their parent genes. An analysis of GO categories and KEGG pathways was done on 3,718 host genes of 33,990 lncRNAs and 4,524 host genes of 22,854 circRNAs to investigate their possible roles in common carp. For the lncRNAs in cellular component category, the top three largest groups were the nucleus (7.31%), plasma membrane (5.85%), and membrane (2.62%); for the biological process: anatomical structure development (10.02%), signaling (8.02%), and protein modification process (5.69%) and for molecular function: transferase activity (7.29%), catalytic activity (5.38%), and hydrolase activity (3.78%). The significantly enriched KEGG pathways were mainly necroptosis, NOD-like receptor signaling pathway, hypertrophic cardiomyopathy, small cell lung cancer, MAPK signaling pathway, pathways of neurodegeneration, and axon guidance (Supplementary Tables S7, S8). The analyses of GO and KEGG indicate a strong potential for lncRNAs and circRNAs to play various roles in biological processes within the common carp fish.

### 3.10 Web resource for common carp lncRNAs and circRNAs

**FIGURE 6:**
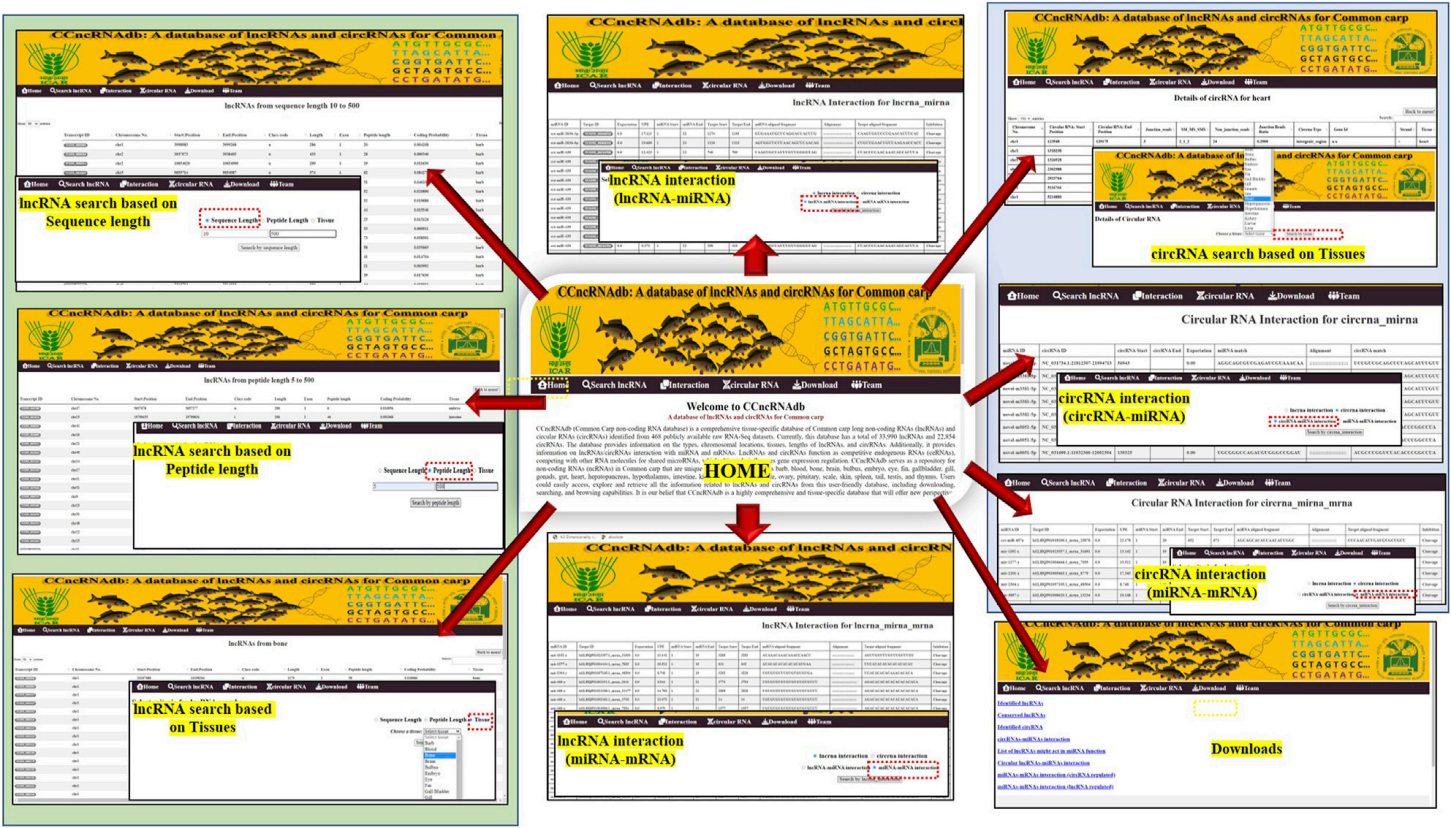
Different interfaces of CCncRNAdb web-resource.

A web-based database, named *CCncRNAdb* for common carp ncRNAs is accessible at http://backlin.cabgrid.res.in/ccncrnadb/index.php, which contains information on 33,990 lncRNAs and 22,854 circRNAs, including their characterization, and interactions with miRNAs and mRNAs. *CCncRNAdb* has six tabs, *namely*, Home, lncRNA, Interactions, circRNA, Download, and Teams (Figure 6). The main features of this database are: *CCncRNAdb* offers extensive information on tissue-specific lncRNAs and circRNAs, such as their chromosome locations, sequence length, and coding potential. To obtain details regarding the lncRNA/circRNA-miRNA-mRNA interaction network, users can visit the “Interaction” tab. The results can be downloaded directly for all the tissues.

## 4 Discussion

The research is oriented towards the tissue-wise studies of long non-coding RNAs and circular RNAs in common carp from the retrieved RNA-seq data from NCBI, followed by tissue-specific/tissue-wise analysis. The tissue-specific identification and ceRNAs analysis in common carp ncRNAs is very limited as compared to humans or other fish species (Nielsen et al., 2014; Chen et al., 2018; Zhang et al., 2020). Previous research on common carp is primarily focused on genes, and miRNAs, with very limited developmental stage-specific lncRNAs. However, the rising importance of non-coding RNAs (ncRNAs), including miRNAs, lncRNAs, and circRNAs, relates to their critical roles in gene expression networks, particularly immune regulation and other biological processes. Possible mRNAs and ncRNAs related to immune regulation were used to create lncRNA-miRNA-mRNA and circRNA-miRNA-mRNA interaction networks. Exploring tissue-specific non-coding RNA identification in common carp is crucial, and this study provides the most comprehensive analysis of lncRNAs and circRNAs using RNA-seq data. To the best of our knowledge, this is the first report that methodically distinguishes lncRNAs and circRNAs based on tissue-wise RNA-seq data.

The 33,990 putative lncRNAs and 22,854 circRNAs across 468 RNA-seq datasets in 28 tissues of common carp fish used in this study were categorized into three groups, based on their proximity to protein-coding genes. Similar to the comparative research on other organisms, the identified putative lncRNAs had lower expression levels, shorter transcript lengths, and fewer exons compared to protein-coding genes (Al-Tobasei et al., 2016; Ning and Sun, 2021a; Chen et al., 2021). Kidney tissue was observed to have the most abundant lncRNAs (23.54%), while the least was seen in bulbus tissue (0.29%). Almost 65% of identified lncRNAs were intergenic (lincRNAs) which was in concordance with the other vertebrate species. Our results revealed distinctive features of identified lncRNAs compared to mRNAs, including reduced exon count, shorter transcript lengths, lower GC content, and lower conversion rates which are in consistent with prior findings (Niazi and Valadkhan, 2012; Al-Tobasei et al., 2016; Bakhtiarizadeh and Salami, 2019). The lncRNA expression value (FPKM) specifically tissue-wise, the details of which are provided in Supplementary Table S3.

Our study on miRNA-lncRNA/circRNA interactions in common carp supports the notion of lncRNAs acting as miRNA targets, limiting their mRNA regulation, which is called lncRNA sponge or competing endogenous RNA (Salmena et al., 2011). The results of a study conducted on mice revealed that miR-338-3p can directly target the SRY-box transcription factor 4 (SOX4) in ESCC cells. The study also showed that SNHG17 can act as an endogenous “sponge” by competing with miR-338-3p to regulate SOX4, consequently promoting tumor progression. These findings suggest that targeting these molecular interactions could serve as a potential therapeutic intervention for ESCC. In the same way, our study found that lncRNA TCONS_00239471 is targeted by miRNAs mir-338-y and ccr-miR-338 so its function will be like therapeutic targets for ESCC (Chen et al., 2021)

Multiple studies have provided evidence that lncRNAs can serve as targets for miRNAs, thereby inhibiting the interaction between miRNAs and coding genes (Paneru et al., 2016; Pereiro et al., 2020). In recent times, there has been evidence to support crucial role of lncRNAs in regulating innate antiviral responses in teleost fish, for example, MARL operates as a ceRNAs for miR-122, thereby controlling the quantity of mitochondrial antiviral signaling proteins (MAVS) and impeding the replication of SCRV while stimulating antiviral responses (Anderson et al., 2015). We also found mir-8499-y to target two lncRNAs, i.e., TCONS_00413548, TCONS_00007855, and six mRNAs to mediate the role in regulating a wide range of cellular processes.

The investigation into tissue-specific circRNAs in common carp gave 22,854 circRNAs and GO analysis suggests that lncRNAs/circRNAs play a crucial role in various cellular processes, including transcriptional regulation, signaling pathways, and enzymatic reactions, which could have implications for various biological processes, including development, growth, directly involved in immunity, and disease (Xiu et al., 2021). For identification of circRNAs on common carp, CIRI2 was used owing to its robustness and reliability as compared to other methods, based on numerous literature (Gao et al., 2018; Chen et al., 2022; Kumar et al., 2023; Rbbani et al., 2023). In tilapia fish, ten and eleven circular RNAs were predicted to target miR-221 and miR-222, correspondingly. One of these, Oni_circRNA_002834, has the capability to bind with miR-221, miR-222, and miR-734, which consequently target certain mRNAs. It has been proposed that alterations in these miRNAs, due to bacterial invasion, may modify the expression of immunomodulatory proteins in tilapia’s brain, potentially enhancing the immune response through an alternative mechanism. In the case of rainbow trout’s circRNA-miRNA-mRNA network found that circRNA5279 and circRNA5277 co-expressed with tap2 via competitive binding with oni-mir-124a-2-p5_1ss13GA. Mir124a regulates T cell activation and differentiation and is critical, having a crucial role in rainbow trout’s skin immunity (Htet and Tennyson, 2016; Zhao et al., 2017). Similarly, our study shows that miRNA mir-6627-y targeted a total of 7 mRNAs, miRNA mir-6651-x targeted 9 circRNAs and 2 mRNAs, and another miRNA mir-7371-x targeted a total of 13 circRNAs so the function also is related to immunity. In this study, the enriched KEGG pathways included necroptosis, NOD-like receptor signaling, hypertrophic cardiomyopathy, small cell lung cancer, MAPK signaling, Fc gamma R-mediated phagocytosis, neurodegeneration pathways, axon guidance, cellular senescence, and more. In zebrafish, KEGG analysis showed processes related to viral infections like endocytosis, MAPK signaling, herpes simplex infection, and NOD-like receptor signaling (Valenzuela-Muñoz et al., 2019). This implies that lncRNAs and circRNAs in common carp may be involved in the immune response and protecting the host from pathogens and tissue damage. This fish species holds significant ecological importance; however, there is currently no existing genomic resource for it. Furthermore, specific tissue-wise reports on lncRNAs are lacking. Consequently, our pioneering investigation into tissue-wise lncRNAs in common carp is the inaugural effort in this field. The outcomes of this study are poised to benefit forthcoming research endeavors greatly.

This study provides the information of circular RNA for the very first time in common carp fish. The extensive web-resource on common carp lncRNAs in the form of *CCncRNAdb*, is freely accessible at http://backlin.cabgrid.res.in/ccncrnadb/index.php which catalogues common carp specific lncRNAs and circRNAs and their interaction studies. This resource will aid in comprehending the fundamental roles that these lncRNAs and circRNAs perform in the growth, development, and response to diseases in common carp. The annotation of the common carp reference genome has been significantly enhanced by the detection of lncRNAs and circRNAs. These putative lncRNAs and circRNAs can aid in improving our comprehension of the biological mechanisms governing regulatory interactions involving mRNA, miRNA, and lncRNA/circRNA.

## 5 Conclusion

This study involves 468 RNA-seq datasets across 28 tissues of common carp for the identification of tissue-specific lncRNAs and circRNAs, and their interactions with miRNAs and mRNAs. A total of 33,990 lncRNAs and 22,854 circRNAs were recognized and characterized. The analysis of the conservation of the identified lncRNAs confirms that lncRNAs are poorly conserved in nature. This study discovered that 19 distinct lncRNAs serve as precursors for 9 miRNAs, which may help in understanding the complex mechanisms of gene regulation. Through GO and KEGG analyses, tissue-specific lncRNAs/circRNAs revealed multiple signaling pathways including necroptosis, NOD-like receptor signaling, hypertrophic cardiomyopathy, small cell lung cancer, MAPK signaling, etc., these findings enhance our comprehension of common carp fish’s evolution, augmentation, and immune system, shedding light on the role of lncRNAs and circRNAs in immune response and their impact on common carp growth and development. The freely accessible *CCncRNAdb* will provide information about lncRNAs and circRNAs in common carp establishing a robust platform for further exploration of lncRNA/circRNAs tissue-specific mechanisms and functions in this species for better management.

## Data Availability

The datasets presented in this study can be found in online repositories. The names of the repository/repositories and accession number(s) can be found in the article/Supplementary Material.

## Supplementary material

Supplementary Figure 1 Secondary structures of **(A)** lncRNA TCONS_00417363 (dark green), and precursor form of the miRNA, ami-mir-133a-2 (color in red); **(B)** lncRNA TCONS_00239471 (dark green) with target locations for two distinct miRNAs, ccr-miR-338 (red) and mir-338-y (blue).

Supplementary Figure 2 Relationship between ceRNA networks where blue rectangle represent lncRNAs, red diamond represents mRNAs and light green ellipse represent miRNAs.

Supplementary Figure 3 Relationship between ceRNA networks, where lncRNAs are denoted by blue rectangles, mRNAs by red diamonds, and miRNAs by light green ellipses.

Supplementary Table 1 Tissue-wise details of the datasets used in the study from different varieties of common carp.

Supplementary Table 2 Tissue-wise summary of sequence reads and alignment statistics from different varieties of common carp.

Supplementary Table 3 Tissue-wise identified lncRNAs from common carp and their characteristic information.

Supplementary Table 4 Conserved lncRNAs among the identified common carp lncRNAs.

Supplementary Table 5 LncRNAs acting as precursors of miRNAs.

Supplementary Table 6 Tissue-wise identified circRNAs from common carp.

Supplementary Table 7 KEGG Pathways associated with common carp lncRNAs.

Supplementary Table 8 KEGG Pathways associated with common carp circRNAs.
