# Supplementary figures and images for: Genome-wide identification and characterization of tissue specific long non-coding RNAs and circular RNAs in common carp (Cyprinus carpio L.)
Source: Front Genet. 2023 Nov 28;14:1239434. doi: 10.3389/fgene.2023.1239434 (PMC10713812; doi:10.3389/fgene.2023.1239434)

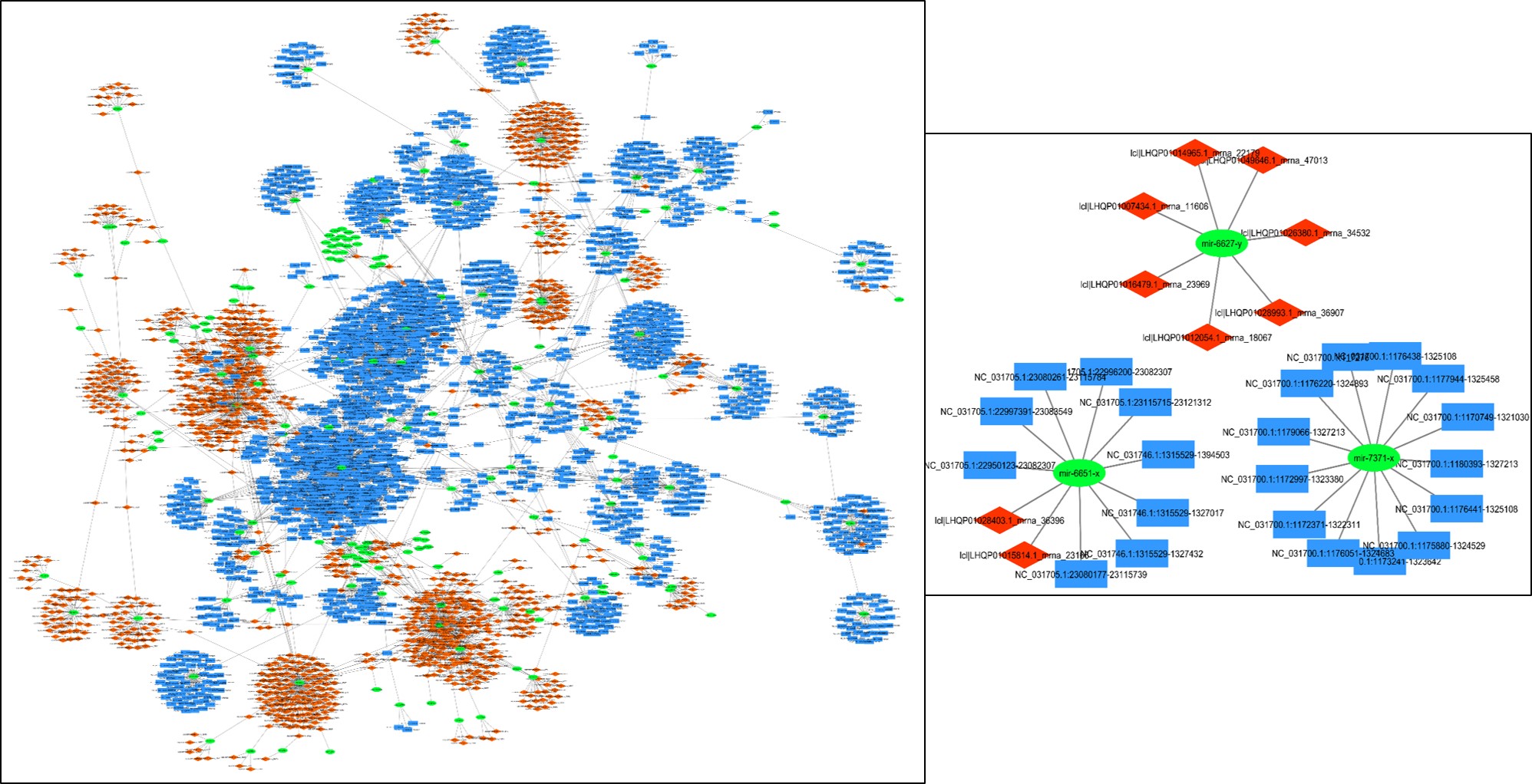

Supplement: Supplementary file 2 [file Image3.jpeg]

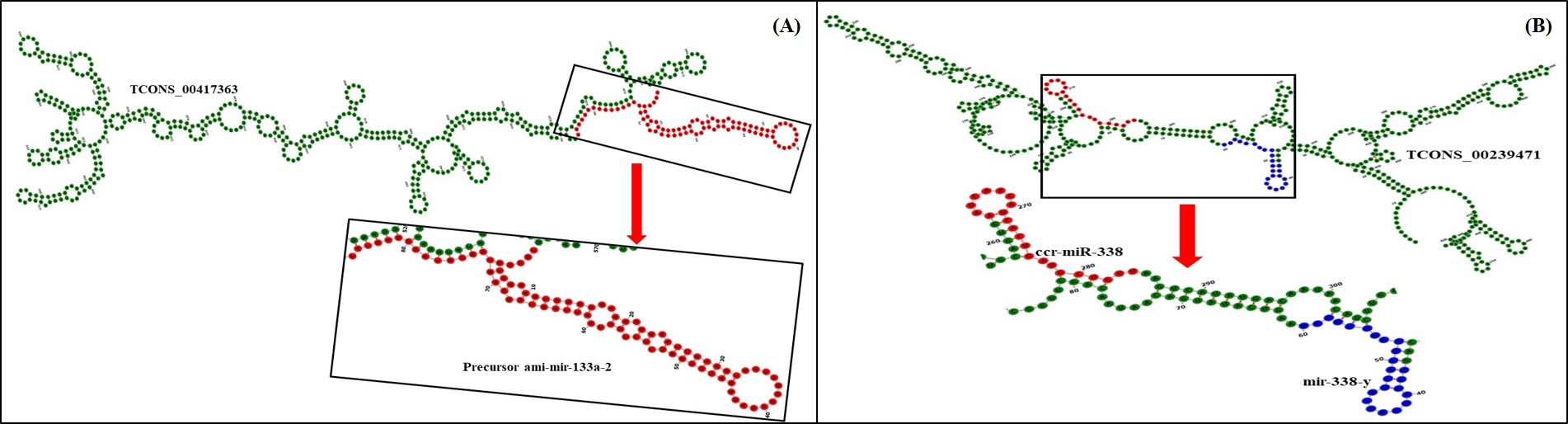

Supplement: Supplementary file 4 [file Image1.jpeg]

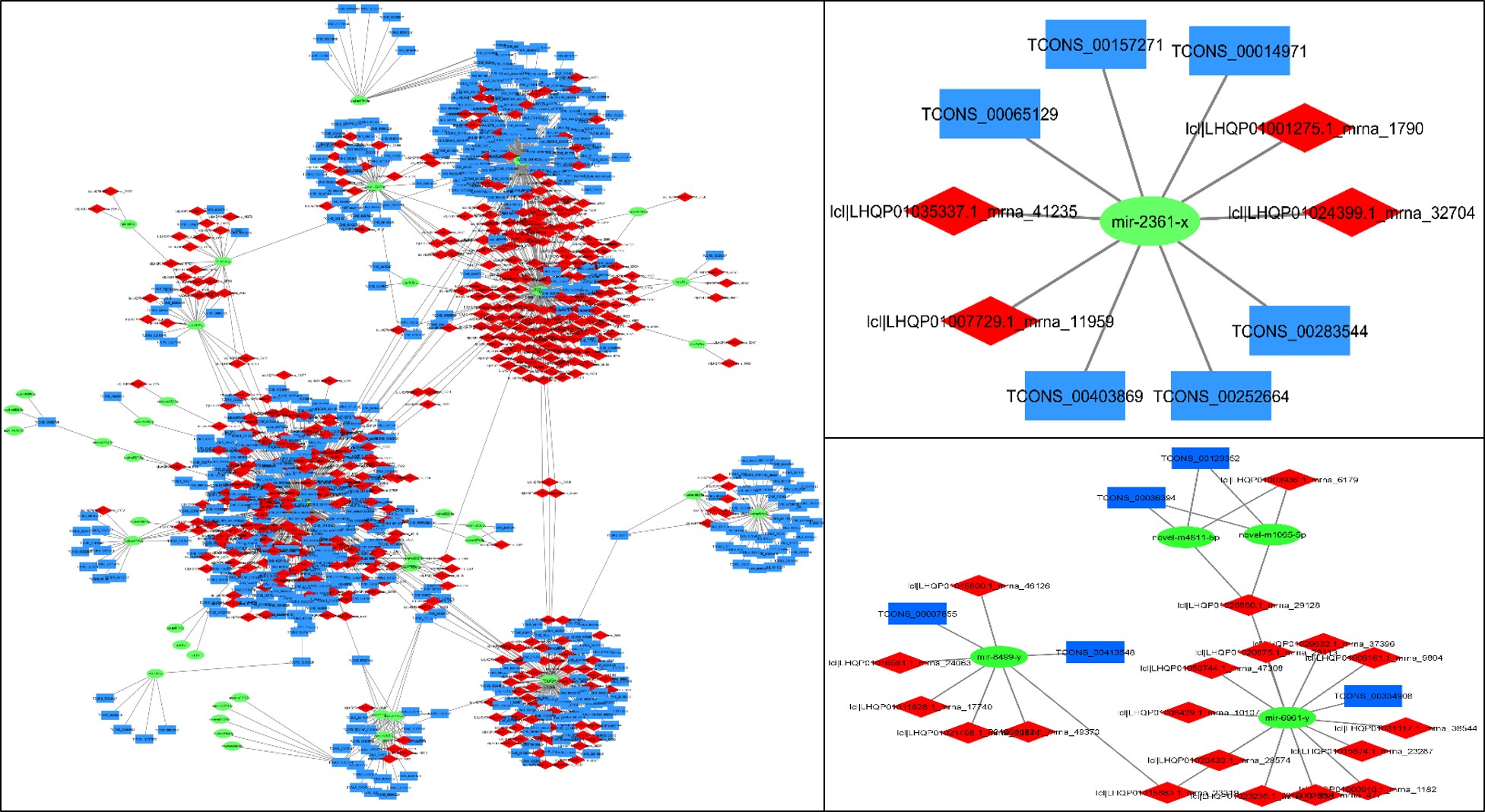

Supplement: Supplementary file 5 [file Image2.jpeg]
